# Longitudinal idiographic assessment of adolescent-dog relationships and adaptive coping for youth with social anxiety: The Teen & Dog Study protocol

**DOI:** 10.1371/journal.pone.0333190

**Published:** 2025-10-07

**Authors:** Megan K. Mueller, Erin K. King, Linda Charmaraman, Jasmine Mote, Eric C. Anderson, Seana Dowling-Guyer, Nicole Mason, Jordanne J. N. Brown, Evan C. Mingo, Rachael A. Sabelli, Emily McCobb

**Affiliations:** 1 Department of Clinical Sciences, Center for Animals and Public Policy, Cummings School of Veterinary Medicine at Tufts University, Grafton, Massachusetts, United States of America; 2 Wellesley Centers for Women, Wellesley College, Wellesley, Massachusetts, United States of America; 3 Sargent College of Health & Rehabilitation Sciences, Boston University, Boston, Massachusetts, United States of America; 4 Center for Interdisciplinary Population and Health Research, MaineHealth Research Institute, Portland, Maine, United States of America; 5 Tufts University School of Medicine, Boston, Massachusetts, United States of America; 6 Department of Medicine and Epidemiology, One Health Institute, University of California, Davis School of Veterinary Medicine, Davis, California, United States of America; PLOS: Public Library of Science, UNITED KINGDOM OF GREAT BRITAIN AND NORTHERN IRELAND

## Abstract

The Teen & Dog Study is a longitudinal research project aimed at understanding the impact of youth-dog relationships on youth coping with social anxiety. The study will follow 514 United States adolescents (ages 13–17) with high social anxiety who live with dogs and their families, collecting longitudinal assessments of their physiological, emotional, and social well-being. With a focus on identifying the mechanisms by which youth-dog interactions may support adaptive coping, the study has three primary aims: (1) assess how the youth-dog relationship contributes to coping with social anxiety over time, factoring in individual, family, and peer influences; (2) investigate family-level processes that enhance youth-dog relationships and identify barriers to optimization; and (3) examine how dog interactions influence adolescents’ physiological responses, particularly in relation to anxiety. The study integrates quantitative and qualitative data, including surveys, interviews, ecological momentary activity, and continuous physiological monitoring, to assess strategies for optimizing youth-dog interactions in the context of social anxiety. This paper outlines the study protocol and presents characteristics of the study sample at baseline. Ultimately, the Teen & Dog Study seeks to inform interventions that harness the benefits of youth-dog relationships to improve mental health outcomes.

## Introduction

Supporting adolescent mental health is a critical and timely issue, with teenagers experiencing high rates of mental health challenges that were exacerbated by the COVID-19 pandemic [1]. Social anxiety is of particular relevance for adolescents, as social anxiety disorder most frequently develops during adolescence [2] and is linked to a host of maladaptive outcomes [3,4]. Recent research has demonstrated that relationships with companion animals, and dogs in particular, can attenuate the effects of social stressors and anxiety for youth [3,5], and may promote specific behavioral and cognitive strategies associated with adaptive coping. The aim of this study is to assess the potential of youth-dog interaction in supporting adaptive coping and thriving for adolescents with social anxiety.

### Social anxiety in adolescence

Social anxiety is a significant mental health challenge for adolescents. Adolescence is generally defined as the age range of 10–19 years [6] with social anxiety developing more frequently during the middle adolescent years (i.e., 13–17 years) [7,8]. Social anxiety disorder is the most common anxiety disorder in the United States, with a lifetime prevalence rate of 7–13% [5,9]. Social anxiety manifests as extreme fear of negative evaluation by others in social settings and avoidance of social interaction [10,11]; it involves a pervasive experience of heightened anxiety and behavioral avoidance in social and performance situations, with an overestimation of negative evaluation by others and/or a failure to appropriately process positive social feedback. Social anxiety is linked to a host of maladaptive developmental outcomes, including other anxiety disorders, depression, and substance use [9,12,13], and there is a strong, reciprocal positive association between social anxiety and loneliness [14].

Recently, the COVID-19 pandemic has further exacerbated rates of social anxiety through social isolation and lack of access to peers, as emerging research is showing that anxiety (including social anxiety) and depression have increased for youth during the pandemic [15]. Youth with social anxiety often lack access to mental health services [16], such that the vast majority (approximately 90%) of those under age 18 don’t receive social anxiety treatment [17]. Given that social anxiety generally begins during childhood or adolescence [18,19], and that untreated social anxiety during childhood and adolescence often persists into adulthood [20], adolescence is a particularly important developmental period for intervening and preventing the negative sequelae that can result from social anxiety.

### Dog interactions and adaptive coping

Identifying and optimizing resources within the family system, such as companion animals, to reduce the risk associated with social anxiety has the potential to significantly impact adolescent quality of life. One area of particularly high clinical and practical value is identifying how to promote adaptive coping to help adolescents manage social anxiety. Adaptive coping includes strategies for managing the effects of a stressor in productive ways such as problem solving or seeking out positive activities as a distraction (versus maladaptive coping, such as substance use), and relies on both behavioral and cognitive strategies [21].

**Fig 1:**
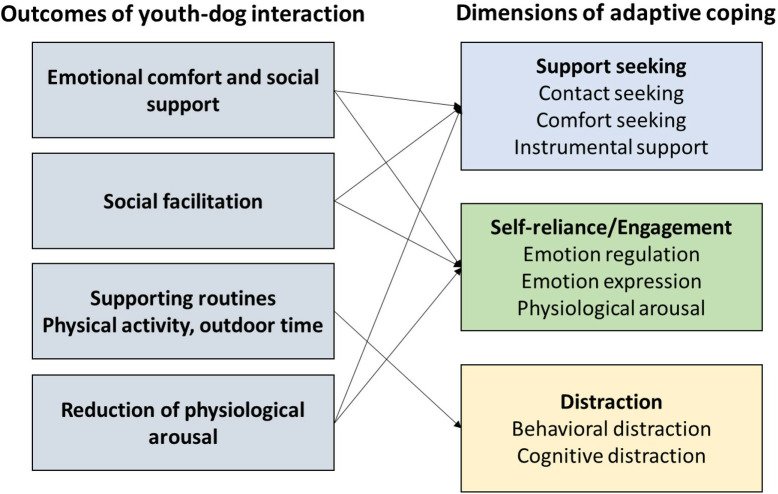
Conceptual model of how adolescent-dog interactions support dimensions of adaptive coping.

Pet dogs are common within many families; recent estimates show that 58 million households in the United States have a dog [22], suggesting that many people, including youth, have regular and frequent interactions with pet dogs. Some of the key outcomes of dog interactions identified in prior research link directly to behavioral and cognitive strategies associated with adaptive coping, such as support seeking, self-reliance/engagement, and positive distraction [23,24] (see Fig 1 for conceptual model). For example, youth frequently report seeking support from their pets when distressed [25], suggesting that this relationship provides comfort, contact, and emotional support. Through the development of emotionally supportive bonds, interaction with a pet has been shown to foster social skills and prosocial behaviors [26–28], which are associated with decreases in anxiety symptoms [29–31]. Through maintaining regular care routines such as feeding, walking, spending time outside, and engaging in physical activity [32,33], dog interactions also support healthy behaviors as an element of positive distraction coping.

Similarly, dogs can also function as a catalyst for facilitating social interactions between people [34,35], thereby strengthening the social context within a family or community and facilitating adaptive coping through social and instrumental support. For example, taking a dog on a walk can be a way that teenagers see and interact with other community members in their neighborhood in a low-stakes manner. Since socially anxious adolescents are unlikely to self-disclose feelings of social discomfort to others [16], finding ways to promote positive emotion expression is an essential component of fostering self-reliance/engagement dimensions of adaptive coping strategies. Particularly in the context of adversity and stress, pets are often perceived as being non-judgmental and are therefore able to provide a comfortable avenue for emotion expression [36].

Dogs can also contribute to the reduction of sympathetic physiological arousal. Hyperarousal is a key feature of social anxiety [37], and the reduction of sympathetic arousal can assist with coping via emotion regulation and reduction in appraisal of stress. Recent evidence has shown that during a social stressor, a pet dog can reduce perceived social stress [5] and improve positive affect [3,38]. Further, emerging evidence indicates that interacting with an animal can attenuate physiological responses related to stress by impacting the hypothalamic-pituitary-adrenal axis cascade in ways that support adaptive responses through support and appraisal modulation [39], as well as physical touch [40–42].

### Design considerations for research on dogs and adolescent social anxiety

Prior research on pets and mental health has produced mixed outcomes [43]. Overall, existing evidence within the field of human-animal interaction (HAI) supports the hypothesis that dog relationships can foster the cognitive and behavioral elements of adaptive coping outlined in Fig 1. However, some of the existing research on companion animals and youth mental health more broadly has found mixed or null relationships between pet ownership and adolescent mental health [44,45]. These contradictory findings suggest complexity in the link between pets and youth mental health that has not been fully explored due to methodological limitations of prior work. To begin with, many studies have relied on pet ownership as a dichotomous predictor, typically for reasons of feasibility [46,47]. Measuring whether or not someone has a pet (regardless of species, when that pet entered their life, or whether they had agency in the decision to obtain said pet) does not account for variability in relationship quality, emotional support, or frequency of interaction, and these features of relationship quality may significantly impact any potential outcomes associated with companion animals [27,34,48]. There is also clear evidence for species-level differences in the effects of pet ownership, with stronger evidence for the role of dogs (vs. other types of pets) in supporting mental health and well-being [49,50].

To date, much of the existing research on these physiological responses has focused on contact with therapy dogs in structured settings [51,52]: these involve interacting with both an unfamiliar therapy dog handler and an unfamiliar dog, a very different setting than interactions in daily life with familiar pet dogs. The relational nature of interacting with one’s own dog in a familiar setting in daily life may have a more significant impact on adaptive responses to physiological arousal than contact with a novel therapy dog. Furthermore, much of the existing research in this area is cross-sectional [53,54], which creates additional limitations in understanding developmental patterns – both quantitatively and qualitatively – and in establishing causality. Therefore, longitudinal studies capitalizing on mixed methods measurement approaches are needed to understand with precision for which youth, and under what circumstances, pet relationships support adaptive coping for youth with social anxiety.

### The Teen & Dog Study

The Teen & Dog Study is a five-year, longitudinal research study assessing the effects of dog relationships on youth with social anxiety and the processes and mechanisms underlying these effects. The Teen & Dog Study will collect physiological, emotional, and social assessments of 514 youth living in the United States and their parent/guardians through a mixed-methodological approach. A baseline cohort of 514 youth between the ages of 13–17 who owned a dog was recruited during a one-year period from 2024–2025. The Teen & Dog Study has three main study aims:

#### Aim 1: Identify inter-individual differences in longitudinal trajectories of associations between adolescent-dog relationships and adaptive coping with social anxiety.

In this aim, we will quantitatively assess how youth-dog relationships contribute to adaptive coping over time, and how individual, family, and peer factors may influence these relationships by collecting quantitative survey data from over 500 youth and their parents. We hypothesize that **1.1)** support from a dog relationship will be associated with positive quantitative trajectories of adaptive coping, and **1.2)** the relationship between support from a dog and adaptive coping will be moderated by individual sociodemographics and peer and family relationship quality.

#### Aim 2: Identify the family-level processes involved in adolescent-dog relationships that support adaptive coping with social anxiety and how these processes change over time.

The goal of this aim is to understand how dogs function within the family system in ways that support both adaptive and maladaptive coping, and what types of family-level processes are involved in mutually-influential youth-dog relationships (both positive and negative). To achieve this aim, we are collecting qualitative interview data from a subsample of 40 youth and their parents at two time points, two years apart. These data will be integrated with Aim 1 survey data to conduct mixed methods analyses identifying the family-level processes involved in adaptive youth-dog interactions and how these processes change over time, to inform how family-based approaches can be optimized.

#### Aim 3: Examine idiographic relationships between affect, anxiety, and arousal in human-dog interactions.

To achieve this aim, we are collecting continuous measures of electrodermal activity (an index of sympathetic nervous system activation) using a wearable wristband, combined with ecological momentary assessment (EMA) of emotions and behaviors. Data will be collected from 100 youth during two week-long periods spaced approximately one year apart, with an additional 50 youth completing a third week-long period to further assess longitudinal patterns. We hypothesize that youth-dog interactions will be associated with **3.1)** immediate reductions in physiological arousal and anxiety, and **3.2)** reduced loneliness and less negative affect.

## Materials and methods

**Fig 2:**
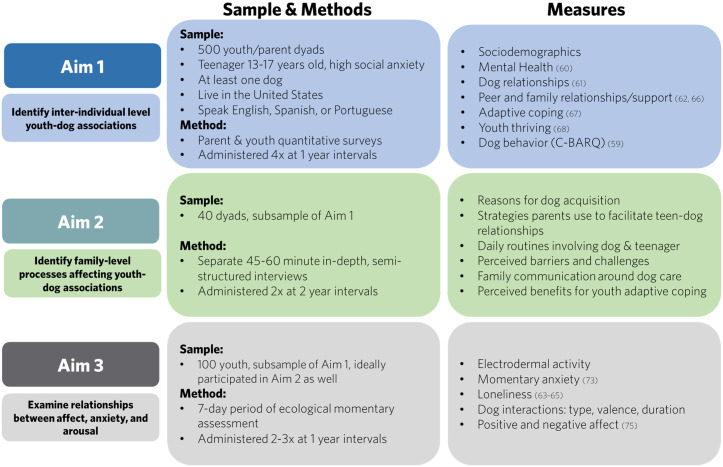
Teen & Dog Study sample, methods, and measures across aims.

In order to understand youth-dog relationships within a complex developmental system, we are using a multi-method, integrated approach to address our three study aims over a five-year period (See Fig 2 for overview of sample, methods, and measures).

### Status/Study timeline

The Teen & Dog Study recruited a baseline sample of parent-youth dyads from February 20, 2024 to February 2025 (Aim 1). Longitudinal data collection is ongoing for Aims 1–3 (Fig 2). Participant recruitment into all three aims will be completed by July 2027 (estimated) and data collection for the entire study completed by July 2028 (estimated). Final results will be expected by December 2028.

### Participants

Participants in the Teen & Dog Study include 514 youth aged 13–17 years and 514 parents or guardians. These 514 participant dyads were yielded from 943 dyads who completed an initial screener survey, of which *n* = 574 were eligible. Adolescents were eligible to participate in the study if they had at least one dog who lived with them in the household where they spent at least 50% of their time, and screened as having high social anxiety based on the Social Anxiety Scale for Adolescents (SAS-A; high anxiety is defined by scores of 50 or higher; [55]). In addition, participants needed to speak and read in English, Spanish, or Portuguese, have one parent/guardian willing to participate, and had to live in the United States. We did not exclude participants based on co-morbid diagnoses, engagement in active treatment with a mental health professional, or medication use, but are tracking this information over time to enable assessing how mental health diagnosis or treatment may impact youth-dog relationships over time. Participants who age into adulthood during the study will continue to be followed (after reconsenting as adults), as it is scientifically valuable to assess the role of animal companionship through this transition to young adulthood. We will also continue to follow families who experience the loss of a dog (e.g., through death), whether or not they acquire another dog during the study period.

Aim 2 of this study includes a subsample of 40 youth and their parent/guardians from the Aim 1 study sample. Based on the Aim 1 survey, families will be selected for participation along a range of sociodemographic variables to ensure that the Aim 2 subsample is representative of the larger study sample.

Aim 3 will include a subsample of 100 youth participants. To maximize the presence of multiple types of data across individuals, we will prioritize enrolling the youth participants who will have data for both Aims 1 and 2 to participate in Aim 3. For the additional participants, the same purposive sampling criteria from Aim 2 will be used to select additional participants for Aim 3.

#### Recruitment.

A multipronged, rolling recruitment approach was used to recruit the sample, beginning in February 2024. To access a national sample that represented geographic, economic, and racial/ethnic diversity, we utilized several recruitment companies. User Interviews, a panel company that targets audiences with a wide range of demographic, professional, behavioral, and technical criteria [56], recruited potential participants using their diverse network of families registered on their platform. In addition, we also worked with the recruitment company CliniContact [57] to distribute study recruitment materials to their network of schools throughout the United States which was particularly helpful in recruiting hard-to-reach populations, such as youth living in a rural setting.

Additionally, participants were recruited by leveraging existing youth/parent contact lists and longitudinal samples. We recruited from an existing longitudinal sample of diverse adolescents via school-based sampling [44] to participate in this study. We also contacted via email youth and parent/guardians who had previously agreed to be contacted about future studies conducted by the investigators’ research labs.

Finally, we recruited participants from youth-serving groups, community events, and libraries and community centers. Members of the study team attended community-led events and visited schools throughout New England to hand out flyers to recruit youth and parent participants. In addition, paper flyers with study information and a QR code for recruitment were distributed throughout libraries and towns in various locations on the east coast of the United States. We also contacted youth programs and libraries/community centers across the country to distribute study information digitally. This method allowed us to reach geographic areas that were underrepresented in our other recruitment methods.

### Participant screening

Interested youth and their parents/guardians were sent a screening questionnaire administered through the REDCap electronic data capture tools hosted at Tufts Medicine [58]. Materials were available in English, Spanish and Portuguese. This questionnaire screened for eligibility criteria, including high social anxiety using the SAS-A [55], as well as collected sociodemographic and dog-related information. Parental consent and youth assent for completing the brief online screening tool were obtained digitally. If the youth/parent dyad were eligible, they were invited to enroll in Aim 1 of the study and consent for these activities was obtained separately. Measures included in the screening survey are outlined in Table 1.

**Table 1:** Screening survey measures: Eligibility criteria and socio demographics.

Adolescent section	Parent section
	**Eligibility** • Lives in the United States • Pet dog who lives with youth/parent for more than half the time • Teenager is 13–17 years • Speaks English, Spanish, or Portuguese
**Sociodemographics** *(youth self-report)* • Gender identity • Age • Racial/ethnic identity	**Sociodemographics** *(parent self-report)* • Age • Gender identity • Racial/ethnic identity • Family composition • Housing type (urban, suburban, rural) • Housing location: zip code • Household income • Highest level of education completed
**Dog** • Name of dog • Role of dog in household (pet, service/assistance animal, emotional support animal, etc.)	
**Social Anxiety** • Social Anxiety Scale for Adolescents SAS-A [55]	

### Aim 1: Quantitative survey

#### Procedure.

For Aim 1, eligible youth participants and their parents/guardians were asked to participate in a longitudinal quantitative survey that is administered once a year, for four years as an online survey through the Tufts Research Electronic Data Capture (REDCap) platform [58]. To consent, the parent was sent a link to an online consent form. After providing informed consent for their own participation as well as their child’s participation, they were directed to the Year 1 survey. After parental consent, youth were sent a separate link to complete an online assent form. After completing their assent form, they were directed to the Year 1 survey. Consent administration via phone was also provided. Questionnaires and associated consent materials were available in English, Spanish, and Portuguese. If a youth participant turns 18 during the course of the longitudinal study, they will be asked to re-consent for their own participation.

#### Measures.

The Year 1 survey asked youth and parent participants about their pets, dog interactions, peer/friend/family relationships, health behaviors, adaptive coping strategies and demographic information which took participants approximately 15–25 minutes to complete (see Table 2 for description of survey measures, which will be repeated longitudinally). Both youth and parent participants will receive a gift card of $20 each for completing each annual survey, for a total of $80 per person over the course of the study. After the successful completion of the youth survey, parents will be asked to complete the Canine Behavioral Assessment & Research Questionnaire (C-BARQ) (see Table 2) on their dog’s behavior administered from the University of Pennsylvania [59]. The parents will receive an additional $5 gift card for successful completion of the C-BARQ, as well as the resulting dog behavioral report.

**Table 2:** Aim 1 survey measures.

	Adolescent survey	Parent survey
**DEMOGRAPHICS**		
*Demographics and Mental Health Treatment*	**Depression** (STOP-D)* [60]	Current medication use (parent and youth)^+^ Current mental health diagnoses^+^ Current engagement in mental health^+^ treatment (e.g., psychotherapy)^+^
**PREDICTORS: Dog relationships**		
*Adolescent-Dog Interactions*	**Dog interactions:** Time spent with dog, responsibility for care, activities with dog ^+^ **Youth-dog relationship quality**: Pet Dog Relationship Inventory- Child (PDRI-C) [61]* Barriers: Lack of time, residential location, housing restrictions, dog behavioral challenges.^+^	**Pets in the home**: number, species, time of acquisition ^+^ **Dog interactions**: *(parent reporting on child)* Frequency taking care of dog (feeding, walking, training, cleaning) ^+^ **Youth-dog relationship quality**: *(parent reporting on child)* Pet Dog Relationship Inventory-Parent (PDRI-P) [61]* Barriers: Frequency the time, behavior, or expectations negatively impact interactions with dog ^+^ **Dog behaviors:** C-BARQ [59]* *administered separately*
**MODERATORS**		
*Peer and social relationships*	**Social support**: Friends subscale (grouped with family) of the Multidimensional Scale of Perceived Social Support (MSPSS) [62]* Online Social support: Social support from online companionship ^+^ **Loneliness**: Adapted items from UCLA Loneliness Scale, short form [63–65]*	
*Family Relationships*	**Social support**: Family subscale (grouped with friends) of the Multidimensional Scale of Perceived Social Support (MSPSS) [62]* Parental Monitoring: Awareness and frequency of proximity [66]*	
**OUTCOMES**		
*Adaptive Coping*	**Adaptive Coping:** Responses to Stress Questionnaire- Peer Stress (RSQ-PS-Child) [67]* Substance use: Use of substances to manage anxiety ^+^ **Youth thriving:** Positive Youth Development- Very Short Form (PYD-VSF) [68]*	**Adaptive Coping**: Responses to Stress Questionnaire-Peer Stress (RSQ-PS-Parent) [67]*

Note: + indicates measures developed by the study team, * indicates previously established measures.

### Aim 2: Qualitative interviews

#### Procedure.

In Aim 2, 40 parent-youth dyads will be interviewed independently from each other at two timepoints, approximately two years apart to assess developmental changes during a critical adolescent developmental period of seeking autonomy from parents and reassurance or comfort from their peers and pet*.* Two interview guides (one for youth and one for their parent/guardians) will be used to assess the family-level processes related to adolescent-dog relationships. Consent and assent forms will be obtained via REDCap prior to the interviews taking place. Youth and parents will be interviewed separately and audio-recorded for transcription via a secure Tufts or Wellesley University Zoom account. Each interview will take approximately 45 minutes.

#### Measures.

Through semi-structured interview questions, we will focus on exploring both parent and youth perspectives on (a) how and why families chose to acquire a dog, and if those reasons relate to anxiety coping specifically, (b) the intentional ways in which youth seek out dog contact for coping and why, (c) the strategies parents use to facilitate youth-dog relationships and coping, (d) daily routines involving the dog and teenager, (e) perceived benefits for youth coping, (f) perceived barriers or challenges to positive youth-dog relationships, (g) family communication patterns around care of and interaction with the dog, particularly during stressful periods of time, and (h) the nature of relationships between family members and the dog.

### Aim 3: Ecological momentary assessment (EMA) and psychophysiology

#### Procedure.

To determine how interactions with a pet dog influence peripheral physiology as an indicator of anxious arousal, we will collect continuous measures of electrodermal activity (EDA; an index of sympathetic nervous system activation) [69] using the Empatica Embrace wristband [70], combined with EMA of emotions and behaviors. EMA, also known as the experience sampling method (ESM), is a method where participants are offered the ability to report on their experiences as they occur during their daily life [71]. In Aim 3, there will be a total of 100 youth participants enrolled, in a series of seven-day (one week) data collection periods repeated 2–3 times at one-year intervals.

Participants interested in participating in Aim 3 will schedule a phone conversation with one of our researchers to discuss the study activities, study timeline, and ask questions. Prior to this meeting, participants will be sent consent and assent forms to review via REDCap. After the consent and assent forms are complete and the participant has spoken with a researcher over the phone, they will be shipped a package with an Empatica Embrace wristband, a lab-provided tablet (if needed), an instruction booklet, and a prepaid return envelope and label for the study materials to be returned after data collection is complete.

#### Measures.

***Physiological reactivity:*** To measure psychophysiological reactivity, we are assessing EDA using the Empatica Embrace wristband. The Embrace is a clinical-grade wearable sensor used to collect psychophysiology data. [70]. Participants wear this wristband on their non-dominant hand, 1–2 centimeters above their wrist. The wristband uses sensors on the band to measure skin conductance. The wristband is connected via Bluetooth to the participant’s mobile device through the Empatica Care Lab mobile app where data are uploaded to the study’s Empatica cloud storage. During each seven-day study period, youth are asked to continuously wear the Embrace wristband, excluding periods where they are swimming or charging the wristband.

#### Ecological momentary assessment.

In addition to the Embrace wristband, participants will be asked to complete EMA prompts, digitally recording their activities and emotions on their mobile device via the LifeData app, which is a secure, EMA platform that is designed for use in clinical trials [72]. The data collected through the app include timestamps, allowing for integration with the physiological measurements collected with the Embrace wristband. Using fixed sampling, participants will respond to seven prompts per day at two-hour intervals (e.g., 8am, 10am, 12 pm, 2 pm, 4 pm, 6 pm, 8 pm local time). See Table 3 for measures. For each week-long burst of sampling, adolescents will be compensated with a $100 gift card, plus an additional $25 gift card if they respond to 60% or more of the EMA prompts during the study period.

**Table 3:** Ecological momentary assessment measures.

Dog interactions		
	*Measure*	*Response options*
*Dog Presence*	Current dog presence	Yes or No
	Interactions in the past 2 hours	• less than 30 minutes • 30 minutes to 1 hour • 1 hour to 1 hour 30 minutes • 1 hour 30 minutes to 2 hours
*Dog Interaction*	Activities done with dog within the last 2 hours (e.g., walking, petting, sitting on a couch or bed).	Select all that apply: • Dog was with me on the couch or bed • Walking/Running • Petting • Playing fetch or another game • Riding in the car • Obedience/Training • Dog was in the same room as me • Feeding • Other
	Initiation of interaction (participant or dog)	• Yes, some of the interactions • Yes, all of the interactions • None of the interactions
	Enjoyability of interactions	1 (*not at all*) to 5 (*very enjoyable*).
**Other Interactions & Activities**		
	***Measure***	***Response Options***
Social Interactions	Interpersonal interactions since the previous prompt	Select all that apply: parents, siblings, other family members, friends, school/teammates, teachers or coaches, strangers, my dog, other animals, no one
Physical Activity	Instances of moderate or intense physical activity since the last prompt. (e.g., running, playing sports)	Yes or No
**Outcomes**		
	***Measure***	***Response Options***
Anxiety	**Social Anxiety:** Adapted 3 item scale of perceived momentary social anxiety in teens [73]	1 *(not at all)* to 7 *(very much)*
Loneliness	**Loneliness**: Adapted 4-item version of the UCLA Loneliness Scale, short form [74]	1 *(not at all)* to 4 *(a lot)*
Affect	**Affect:** Six item scale capturing high and low activation of positive (3 items) and negative (3 items) emotions, following the affective circumplex model of emotion [75]	1 *(not at all)* to 4 *(a lot)*

### Safety considerations

This study will be conducted in accordance with ethical standards and has been approved by the Social, Behavioral, and Educational Research Institutional Review Board (SBER IRB) at Tufts University (protocol #03434), with a reliance agreement executed with Wellesley College (via Brandeis University) to collect qualitative interview data. Given the content of the questions and data collection methodology, this study presents minimal risk to participants. If a participant discloses information that they are in danger or may cause injury to self or others, we will follow all procedures outlined by the Tufts University Institutional Review Board for appropriate reporting, as indicated by the nature of the disclosure.

## Planned data analyses

### Data management and storage

Data will be stored in the Tufts REDCap system, a secure, web-based application designed to support data capture for research studies, providing 1) an intuitive interface for validated data entry; 2) audit trails for tracking data manipulation and export procedures; 3) automated export procedures for seamless data downloads to common statistical packages; and 4) procedures for importing data from external sources [58]. De-identified data will also be stored in the Tufts University Box platform, which is similarly protected with two-factor authentication. The Teen & Dog Study is registered on Open Science Framework (DOI 10.17605/OSF.IO/43YT7). De-identified survey data will be shared on OSF after data collection. Raw qualitative interview data will not be posted due to the risk of sensitive or identifying information, however, excerpts will be made available to researchers upon request to the corresponding author.

### Aim 1 analyses

Data analysis plans for Aim 1 include longitudinal analyses using Structural Equation Modeling (SEM) techniques to assess patterns of change in both predictor and outcome variables across four times of testing. Longitudinal SEM techniques such as Latent Growth Curve Analysis and Growth Mixture Modeling [76] will allow us to address questions about the impact of dog interactions on youth development. For example, Latent Growth Curve Analysis allows identification of nonlinear trajectories of adolescent adaptive coping; by extension, Growth Mixture Modeling will allow us to determine whether these trajectories are differentially predicted by the four indices of dog relationship quality, and whether/how the moderator variables (e.g., socio demographics, peer relationships, family relationships) impact these relationships.

### Aim 2 analyses

Inter-rater reliability for the independent coders will be established in order to protect against respondent and investigator bias [77]. Independent coders will employ inductive thematic analysis of the transcribed interview dialogue to examine overarching themes, and a second coder who will iteratively code 10% of the data until coding consensus reaches a minimum of 80%. Since we will be conducting serial interviews with the same interviewees over time, we will employ a narrative style analytical approach that involves individual and paired case sets with the aim to achieve saturation of a diverse range of qualitative data. Each adolescent and parent will also be analyzed via dyadic analysis of paired adolescent/parent over two time points. Codes will be based on those developed during the first set of interviews in Phase 1 to be analyzed for change or maintenance of behaviors/attitudes over time [78]. To ensure trustworthiness of the qualitative data analysis, we will incorporate the scholarly literature while the data analysis proceeds by confirming or refining existing theories, resolving coding disagreements by discussion and refining codebooks as needed, obtaining outsider perspectives through a consultant review, and conducting negative case analysis, i.e., returning to the existing data to verify coherence of categories [79]. As inductive thematic analysis is an iterative process, analysis of interview data will begin when the first two interviews have been completed [80]. We will use the latest version of NVivo [81] to facilitate the process of refining the codes, theory development, and visual mapping of the emerging and interrelated concepts.

To analyze our sequential quantitative/qualitative data, we will follow a triangulation protocol, in which we use different methods to gain a more complete picture (rather than only examining corroboration between sets of findings) [82]. Triangulating findings takes place at the interpretation stage of a study after both the quantitative and qualitative data sets have been analyzed separately. Findings from each component of the study will be listed side by side in order to determine where findings from each method agree (convergence), offer complementary information on the same issue (complementarity), or appear to contradict each other (divergence) [83–85] which will lead to a better understanding of our longitudinal findings and highlight where we need to explore further.

### Aim 3 analyses

EDA data will be screened for quality using two different automated procedures. First, Empatica’s built in, proprietary algorithm calculates when the wristband is being worn correctly and is collecting valid data [70]. Second, we will use an approach validated by Kleckner et al. [86] that uses a simple, transparent process for determining EDA quality: EDA out of range, EDA changing too quickly, temperature out of range, and proximity to (within 5 sec) invalid portions based on the first three criteria. Periods of physical activity will be screened for the effects of activity on the EDA analysis. The percentage of usable data, particularly during self-identified interactions with pets, will be used as an indicator of reliability for this measurement method in the context of youth-dog relationships.

Next, we will create standardized EDA reactivity scores for each event of interest (e.g., interactions with pet dogs). These will be created from screened data by subtracting that participant’s baseline EDA level before the event from their EDA during the event of interest. EDA reactivity will be aligned with activity diary data via timestamps from both the Embace and the LifeData app. We will assess changes in EDA reactivity before, during, and after the measurement prompts to examine the ways in which physiological arousal changes in real-time for adolescents, and how this arousal relates to interactions with their dogs and measures of anxiety, affect, and loneliness. To test our hypotheses, we will analyze the micro-longitudinal relationship between EMA-reported instances of dog interactions, loneliness, anxiety, negative affect, and EDA using hierarchical linear modeling (HLM) with robust parameter estimates. For all models, Level-1 variables will be group mean centered (for each participant) and Level-2 variables will be grand-mean centered (across participants).

## Preliminary data

### Participant characteristics

**Fig 3:**
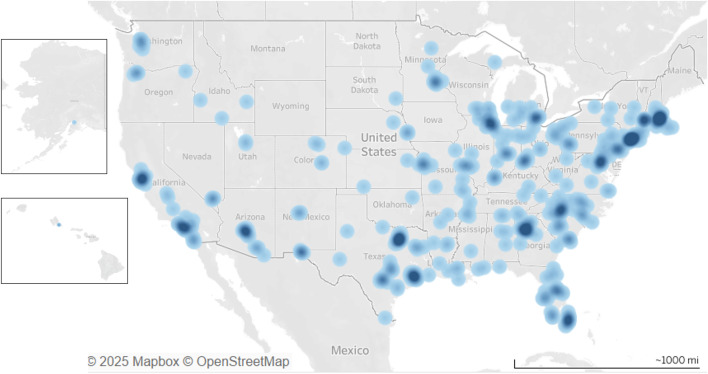
Heat map of participant state of residence.

The baseline cohort of youth/parent dyads (**N* *= 514) is described in Table 4. Youth participants were 13–17 years of age at time of enrollment, with a mean age of 15.6 years (*SD* = 1.3). About 57% (292) of the sample lived in suburban settings, 25% (125) urban, and 18% (92) rural. The majority of parent/guardians in the sample attended college, with 32% (163) having a bachelor’s degree and 25% (129) having a graduate or professional degree. About 1% [6] of parents/guardians completed less than high school, 9% [48] completed high school/GED, 22% (112) had some college and 10% [51] earned an associate’s degree. Participants were geographically diverse, residing in 44 states across the United States; 12.7% of participants were from Texas, 7.8% from California, 7.4% from Massachusetts, 7.2% Florida, 5.1% Georgia, and the remaining states <5% (see Fig 3 for heat map of participant location).

**Table 4:** Demographic characteristics.

	Parent n (%)	Youth n (%)
**Gender identity**		
Woman	441 (85.8%)	317 (61.7%)
Man	68 (13.2%)	164 (31.9%)
Non-Binary	–	9 (1.8%)
Transgender man	–	6 (1.2%)
Genderqueer/genderfluid	1 (0.2%)	5 (1.0%)
Transgender woman	–	2 (0.4%)
Prefer not to answer	–	4 (0.8%)
Prefer to self-describe	–	2 0(.4%)
Race*		
White	357 (69.5%)	361 (70.2%)
Black or African American	109 (21.2%)	131 (25.5%)
Asian	33 (6.4%)	36 (7.0%)
Other Race	25 (4.9%)	30 (5.8%)
American Indian or Alaskan Native	19 (3.7%)	16 (3.1%)
Middle Eastern/North African	4 (0.8%)	2 (0.4%)
Native Hawaiian or Pacific Islander	2 (0.4%)	1 (0.2%)
Prefer not to answer	–	4 (0.8%)
Ethnicity*		
Non-Hispanic	397 (77.2%)	387 (75.8%)
Hispanic – Central/South American	67 (13.0%)	65 (12.6%)
Hispanic – Caribbean	24 (4.7%)	23 (4.5%)
Other Hispanic	20 (3.9%)	30 (5.8%)
Hispanic – European	2 (0.4%)	4 (0.8%)
Prefer not to answer	–	1 (0.2%)

*Racial/ethnic identity groups are not mutually exclusive.

All participants owned a dog as an eligibility requirement of this study. About 63% (288) of the participants lived in a multi-pet household including 33.7% (173) living with a cat, 4.1% [21] with a guinea pig, hamster, or small rodent, 2.3% [12] with a rabbit, 9.1% [47] with a fish, 4.1% [21] with a bird, 7.4% [38] with a reptile, 2.1% [11] with another small animal, 1.2% [6] with a horse, 1.8% [9] with another large animal (e.g., cow, pig, goat), and 1.9% [10] with another type of pet. Dogs in this sample ranged in age from 1 to 16 years with an average age of 5.0 years (*SD* = 3.33), and the average length of time the family had a dog was 4.4 years. Most dogs were spayed or neutered (68.5%, *n* = 352) and vaccinated for rabies (95.9%, *n = *493). The vast majority of participants described their dog in good health (97.3%, *n = *500). Behaviorally, 28.0% (**n* *= 144) of dogs have participated in formal training classes and 79.4% (*n = *408) considered their dog to be comfortable around other dogs.

### Year 1 survey measures

As a preliminary analysis, each scale/measure from the Year 1 survey was assessed descriptively, as shown in Table 6.

**Table 6:** Descriptive statistics of Aim 1 measures.

	*Min*	*Max*	*Mean*	*Median*	*SD*	*Skewness*	*Kurtosis*
PDRI-C: Affection^1^	5.00	25.00	22.56	24.00	3.30	−1.91	4.24
PDRI-C: Nurturance^1^	5.00	25.00	21.42	23.00	3.85	−1.18	1.00
PDRI-C: Emotional Support^1^	5.00	25.00	20.14	21.00	4.42	−.87	.22
PDRI-C: Companionship^1^	5.00	25.00	19.80	20.00	4.36	−.63	−.35
PDRI-C: Friction^1^	5.00	25.00	10.26	10.26	4.20	1.11	1.11
PDRI-C: Pets as Substitute^1^	5.00	25.00	17.72	19.00	5.43	−.40	−.84
PDRI-P: Affection^1^	5.00	25.00	22.15	23.00	3.49	−1.58	2.61
PDRI-P: Nurturance^1^	8.00	25.00	20.05	21.00	4.27	−.65	−.44
PDRI-P: Emotional Support^1^	6.00	25.00	19.46	20.00	4.43	−.72	−.01
PDRI-P: Companionship^1^	5.00	25.00	18.66	19.00	4.90	−.45	−.70
PDRI-P: Friction^1^	5.00	25.00	8.53	8.00	3.28	1.44	3.21
PDRI-P: Pets as Substitute^1^	5.00	25.00	16.30	17.00	5.66	−.22	−.95
MSPSS Family Support^2^	4.00	28.00	22.38	23.00	5.13	−.92	.67
MSPSS Friend Support^2^	4.00	28.00	21.06	22.00	6.03	−.89	.19
Social Media Support^3^	1.00	5.00	2.97	3.00	1.26	−.17	−.95
Loneliness^4^	5.00	20.00	12.01	12.00	3.37	.06	−.56
Parental Monitoring^5^	12.00	25.00	22.16	23.00	2.79	−1.12	.94
Positive Youth Development^6^	33.00	85.00	62.70	63.00	9.67	−.32	−.03
Depression^7^	0	9.00	3.69	3.00	2.56	.31	−.86
**Responses to Stress Questionnaire (RSQ) Coping Subscales**	*Min Ratio*	*Max Ratio*	*Mean Ratio*	*Median Ratio*	*SD Ratio*		
Youth – Primary Control Coping^8^	.08	.28	.17	.16	.04		
Youth – Secondary Control Coping^8^	.09	.37	.22	.21	.05		
Youth– Disengagement Coping^8^	.10	.22	.16	.16	.02		
Youth– Involuntary Engagement^8^	.14	.42	.26	.26	.05		
Youth– Involuntary Disengagement^8^	.09	.27	.19	.20	.03		
Parent – Primary Control Coping^8^	.08	.29	.18	.18	.04		
Parent – Secondary Control Coping^8^	.10	.37	.22	.21	.05		
Parent– Disengagement Coping^8^	.09	.22	.15	.15	.02		
Parent– Involuntary Engagement^8^	.14	.40	.26	.27	.05		
Parent– Involuntary Disengagement^8^	.10	.31	.18	.18	.04		

Note: Possible ranges for measures ^1^PDRI-C/-P: 5–25; ^2^MSPSS: 4–28; ^3^Social Media Support: 1–5; ^4^Loneliness: 5–20; ^5^Parental Monitoring: 5–25, ^6^PYD-VSF: 17–85; ^7^Depression: 0–9, ^8^RSQ: 0–1.

## Discussion and conclusions

The Teen & Dog Study will lay the groundwork needed to design and evaluate specific interventions integrating pet dogs for youth with social anxiety, as well as programs and policies that reduce barriers to dog ownership for diverse families. In addition to scientific publications, results regarding practical applications will be disseminated to mental health practitioners, parents, and educators in community settings.

The results from this study will generate data that will answer key questions about individual specificity in beneficial human-animal relationships that have been previously underexplored. This project will set the stage for future research studies that can explore specific processes and mechanisms and enact interventions that align with precision medicine principles of person-specific care [87]. This study will provide critical information to parents and mental health practitioners about how to support teenagers leveraging their dog as a scaffold for social interactions, and how dogs may be integrated into existing treatment plans for social anxiety. It will also provide useful data for the public regarding reasonable expectations for the benefits of dog ownership.

This is the first study to explore adolescent-dog relationships longitudinally within the context of a family and address the conditions in which dog relationships serve as a protective relationship for youth with social anxiety. By assessing both processes and mechanisms involved in dog interaction and adaptive coping, we will identify methods to maximize adaptive coping strategies supported by dog interactions during a crucial period of adolescent social development. The results will enable evidence-based recommendations for practitioners to leverage dog relationships that can inform future research on interventions involving dogs.
